# Alzheimer’s disease‐associated gut microbes shape neuroinflammatory tone and disease outcomes in mouse models

**DOI:** 10.1002/alz.087048

**Published:** 2025-01-03

**Authors:** Lisa Blackmer‐Raynolds, Maureen Sampson, Adam M Hamilton, Aimee Yang, Anna Kozlov, Lyndsey Lipson, Jianjun Chang, Sean D Kelly, Steven A Sloan, Timothy R. Sampson

**Affiliations:** ^1^ Emory University, Atlanta, GA USA; ^2^ Emory University School of Medicine, Atlanta, GA USA

## Abstract

**Background:**

Changes in neuroinflammatory tone have been shown to modulate neuroimmune responses to Alzheimer’s disease (AD) pathology and shape disease outcomes, however, extrinsic factors that modify neuroimmune activation remain poorly understood. The gut microbiome is one such factor, with the ability to shape peripheral and central immune activation, as well as AD pathologies. AD patients display unique changes in microbiome composition, however, the link between specific AD‐associated gut bacteria, neuroinflammatory tone, and AD outcomes remains to be elucidated.

**Method:**

To identify AD‐associated bacteria that modify neuroinflammatory tone, wildtype germ‐free mice were mono‐colonized with type strains of bacteria species of interest (*Escherichia coli, Bacteroides thetaiotaomicron, Clostridium celatum*, and *Lactobacillus johnsonii*) for 2 weeks. Further evaluation of the effect of *E. coli*—an identified neuroimmune modulatory bacteria—on AD outcomes was performed by exposing conventional 5xFAD mice to *E. coli* via oral gavage for one month. Neuroinflammatory outcomes were assessed by bulk and single cell RNA‐seq and protein analysis. In addition, AD outcomes including cognitive and pathological markers were evaluated in *E. coli* exposed 5xFAD mice.

**Result:**

AD‐associated bacteria induced bacteria‐ and sex‐specific changes in cytokine levels as well as myeloid cell gene expression within the brains of mono‐colonized mice. In particular, *E. coli* was shown to induce a distinct, AD‐associated neuroinflammatory phenotype that was characterized by increased MHC II antigen presentation and changes in boarder associated macrophage and endothelial cell gene expression. Further, *E. coli*‐exposed 5xFAD mice displayed accelerated cognitive decline compared to vehicle controls. Neuroinflammatory and pathological markers in the brains of *E. coli*‐exposed 5xFAD mice were also evaluated to further explore the effects of *E. coli* on AD pathophysiology.

**Conclusion:**

Together, these results highlight the neuroimmune modulatory potential of AD‐associated gut bacteria. In particular, the present study demonstrates how increased intestinal exposure to non‐pathogenic *E. coli* is sufficient to modify neuroinflammatory tone, cognition, and pathology in 5xFAD mice, highlighting the potential importance of this microbe for AD.

